# Matrix Metalloproteinase 2 Contributes to Pancreatic Beta Cell Injury Induced by Oxidative Stress

**DOI:** 10.1371/journal.pone.0110227

**Published:** 2014-10-15

**Authors:** Chongxiao Liu, Xiaoyu Wan, Tingting Ye, Fang Fang, Xueru Chen, Yuanwen Chen, Yan Dong

**Affiliations:** 1 Department of Endocrinology, Xinhua Hospital, Shanghai Jiao Tong University School of Medicine, Shanghai, China; 2 Department of Gastroenterology, Xinhua Hospital, Shanghai Jiao Tong University School of Medicine, Shanghai, China; Broad Institute of Harvard and MIT, United States of America

## Abstract

**Objective:**

To investigate the role of matrix metalloproteinase 2 (MMP2) in pancreatic beta cell injury induced by oxidative stress.

**Methods:**

Rat pancreatic beta cell line INS-1 cells were treated with advanced glycation end-products (AGE) to induce intracellular oxidative stress. Intracellular MMP2 expression and activity were determined by quantitative reverse transcription polymerase chain reaction (RT-PCR), Western blotting, and zymography, respectively. MMP2 expression and activity were manipulated by over-expression with recombinant MMP2 plasmids or knockdown with either MMP2 specific siRNA or inhibitors, and effects on apoptosis and insulin-secretion were measured by flow cytometry and ELISA.

**Results:**

AGE treatment induced intracellular oxidative stress in INS-1 cells, as indicated by elevated ROS levels, apoptotic cell death, and suppressed insulin secretion. This was accompanied by increased MMP2 expression and activity. However, Antioxidant N-acetylcysteine (NAC) treatment inhibited MMP2 expression and activity, and partially reversed cell apoptosis and insulin secretion dysfunction induced by AGE. Forced expression of MMP2 mimicked the effects of AGE treatment while inhibition of MMP2 either by a specific MMP2 inhibitor or MMP2 siRNA protected oxidative stress induced by AGE.

**Conclusion:**

MMP2 expression and intracellular activity are increased by oxidative stress, contributing to cellular dysfunction and apoptosis in INS-1 cells after AGE challenge.

## Introduction

Type 2 diabetes mellitus (T2DM) is generally considered to be caused by the dysfunction of pancreatic beta cells and insulin resistance. Failure of pancreatic beta cells by their progressive loss is regarded as a critical step in the pathogenesis of T2DM [1], [2]. However, the mechanisms underlying pancreatic beta cell dysfunction and loss are still unclear.

Accumulating evidence has indicated that pancreatic beta cell loss in T2DM partially results from oxidative stress [3], [4], [5]. Pancreatic beta cells are more vulnerable to oxidative stress because of low antioxidase activity [6], [7]. The factors responsible for pancreatic beta cell damage that lead to cell dysfunction via oxidative stress remain unknown. It is generally believed that direct chemical modification contributes to that process. Reactive oxygen species (ROS) can oxidize or nitrify proteins, lipids and DNA by direct chemical modification, leading to dysfunction of crucial proteins including signal transduction factors, ribosomal subunits, DNA repair enzymes, and proteases associated with energy metabolism in pancreatic beta cells [8]. Nevertheless, whether factors other than direct chemical modification might contribute to pancreatic beta cell dysfunction in oxidative stress is largely unknown.

Intracellular matrix metalloproteinases (MMPs) including MMP2 are involved in myocardial cell damage via oxidative stress [9]. MMPs belong to a family of zinc-dependent endopeptidases which degrade extracellular matrix and contribute to tissue remodeling in angiogenesis, embryogenesis, atherosclerosis, aortic aneurysm and myocardial infarction [10], [11], [12], [13]. Given that MMP2, a member of the gelatinase family of proteases, also plays vital roles in myocardial ischemia-reperfusion injury [14], it is hypothesized that MMP2 may also have biological functions besides degrading extracellular matrix. Increased MMP2 activity has been reported to result in proteolysis of cytoskeletal proteins including myocardial troponin, myosin, and actin which causes oxidative stress and mitochondrial injury, and ultimately leads to cardiomyocyte dysfunction. Additionally, inhibition of MMP2 activity has been found to attenuate myocardial damage [15], [16]. Similarly, elevated MMP2 has been implicated in the development of diabetes mellitus, and a MMP inhibitor, PD166793, reduces blood glucose in Zucker diabetic rats [17]. However, it remains unclear whether suppression of MMPs activity is associated with remission of pancreatic beta cell damage.

In the present study, we aim to investigate the role of MMP2 in pancreatic beta cell injury induced by oxidative stress.

## Materials and Methods

### 1. INS-1 Cells Culture

Rat pancreatic beta cell line INS-1 cells were donated by Shanghai Institute of Endocrine and Metabolic Diseases and cultured in Roswell Park Memorial Institute (RPMI) medium1640 as previous described [18].

### 2. Cytosolic ROS generation in INS-1 cells

To determine the effects of oxidative stress in intracellular MMP2 expression and activity, INS-1 cells were challenged by an intracellular oxidative stress with Glycated bovine serum albumin (GA), which acts as an Advanced glycation end-products (AGE). The control group was treated with BSA. A third group was co-treated with GA and an antioxidant NAC (Sigma-Aldrich, St. Louis, Missouri, USA) in different concentrations to block intracellular ROS generation.

Cytosolic ROS levels were analyzed by 2′,7′-dichlorodihydroflurescein diacetate (DCFH-DA, Sigma-Aldrich, St. Louis, Missouri, USA) through flow cytometry. The INS-1 cells were washed and incubated with 10 µM of DCFH-DA for 40 min. INS-1 cells were then harvested, washed twice with cold PBS, and directly collected before detection of the mean fluorescence intensity (MFI) of 2′,7′-dichlorofluorescein (DCF) for 10^5^ cells per sample to measure cellular ROS levels (excitation 490 nm, emission 530 nm).

### 3. Inhibition of MMP-2 activity

To determine the protective role of MMP2 inhibition in beta cell dysfunction induced by oxidative stress, INS-1 cells were co-treated with GA and a MMP2 specific inhibitor (cis-9-Octadecenoyl-N- hydroxylamide, OA-Hy, Santa Cruz Biotechnology, Dallas, Texas, USA) for 24 h, the other two groups were treated with GA or BSA.

### 4. Forced expression of MMP-2

A 2.1-kb fragment containing the open reading frame of the rat pro-MMP2 gene was obtained by RT-PCR using the following primers, which contained restriction endonuclease sites XhoI and KpnI. The sense primer was 5′- TCCGCTCGAGATGGAGGCACGATTGGTCTG-3′, and the anti-sense primer was 5′- ATGGGGTACCTCAGCAGCCCAGCCAGTCC-3′. After sequence confirmation, MMP2 was subcloned into the XhoI and KpnI sites of the eukaryotic expression plasmid pcDNA3.1(+) vector. INS-1 cells were transfected with the pcDNA3.1-MMP2 and pcDNA3.1 plasmid. Prior to transfection, INS-1 cells were seeded in 12-well plates at a density of 10^5^/mL and were maintained in medium RPMI 1640 without antibiotics. When the density reached 50%–70%, the cells were grown in Opti-MEM I reduced serum medium (Invitrogen, Life Technologies, Carlsbad, California, USA) during transfection for 6 h. Then, the medium was replaced with normal medium. Transient transfection of INS-1 cells was performed using lipofectimine 2000 reagent (Invitrogen) as per the manufacturer’s instructions.

### 5. Transfection of INS-1 cells with MMP2-siRNA

INS-1 cells were transfected with MMP2 siRNA using a transfection reagent kit (Santa Cruz Biotechnology) according to the manufacturer’s instructions. The transfection complexes, prepared by adding MMP2 siRNA and siRNA transfection reagent, were incubated for 30 min at room temperature, and the cells were incubated with this transfection complex for 8 h at 37°C. Parallel incubations were performed using non-targeting scrambled siRNA. After transfection for 48 h, INS-1 cells were rinsed with PBS and incubated with GA or BSA for 24 h to test the protective effect of MMP2 knockdown in oxidative stress injury. Annealed double-stranded small-interfering RNA (siRNA) for rat MMP2 were designed and synthesized by Genepharma (Shanghai, China) following the sequence 5′-CUGCCUUUAACUGGAGUAATT-3′.

### 6. Determination of MMP2 expression

The mRNA expression level of MMP2 was determined by quantitative reverse transcription polymerase chain reaction (RT-PCR). In brief, total RNA of INS-1 cells was extracted, and cDNA was synthesized from 1 µg RNA with PrimeScript RT Master Mix (TaKaRa, Osaka, Japan). RT-PCR analyses were performed using SYBR Premix Ex Taq (TaKaRa). The standard PCR conditions consisted of 95°C for 30 sec, followed by 40 cycles of 95°C for 5 sec and 60°C for 34 sec, with a final dissociation stage, and the samples were run on an ABI 7500 detector (Applied Biosystems, Foster City, CA, USA). The primer sequences for the rat cells were as follows: Fwd 5′-GAGTAAGAACAAGAAGACATACATC-3′ and Rev 5′-GTAATAAGCACCCTTGAAGAAATAG-3′ for MMP2; and Fwd 5′-GC AAGTTCAACGGCACAG-3′ and Rev 5′-GCCAGTAGA CTCCACGAGAT-3′ for GAPDH.

Protein expression levels of MMP2 were determined by Western blot analysis. Total proteins were extracted using a radioimmunoprecipitation assay (RIPA) lysis buffer and were subsequently separated by 10% SDS-PAGE gels. Proteins were then electrophoretically transferred onto PVDF membranes and probed with the corresponding antibodies (Abcam, Cambridge, MA, USA) overnight at 4°C after blocking in 5% non-fat milk in Tris-buffered saline with Tween-20 (TBST) for 2 h. Secondary peroxidase-coupled antibodies were applied, and a chemiluminescent substrate system was used to detect the signals. Band intensity was analyzed using ChemiDoc XRS+ software (Bio-Rad Laboratories, Hercules, CA, USA).

### 7. Gelatinolytic activity of MMP2

Gelatinolytic activity of MMP2 was measured in INS-1 cells by zymography. Proteins were extracted using the same protocol described above but without denaturation. An equal amount of protein was loaded on the 10% SDS-PAGE gels containing 1 mg/mL gelatin. The gels were equilibrated in the Novex zymogram renaturing buffer (Invitrogen) for 30 min at room temperature with gentle agitation, before being incubated in Novex zymogram developing buffer (Invitrogen) at 37°C overnight. The gels were then stained with Coomassie Blue dye (Invitrogen) and photographed after destaining in double-distilled water for at least 7 h. Band intensity was analyzed by Bio-Rad ChemiDoc XRS+ software.

### 8. Apoptosis of INS-1 cells

Apoptosis of INS-1 cells was detected by flow cytometry using an Annexin V- FITC/PI apoptosis assay kit (Becton, Dickinson and Company, Franklin Lakes, NJ, USA) following the manufacturer’s instructions. Briefly, the cells were harvested, washed twice with cold PBS, and stained with Annexin V-FITC/PI for 15 min in the dark. The cells were then measured by flow cytometry.

### 9. Insulin secretion of INS-1 cells

For each experimental group, INS-1 cells were pre-incubated in Kreb’s buffer containing: NaCl 140 mM, KCl 4.6 mM, MGAO_4_ 1 mM, NaHPO_4_ 0.15 mM, NaHCO_3_ 5 mM, CaCl_2_ 2 mM, and HEPES 30 mM, pH 7.4, and mixed with 0.2% BSA and 3.3 mmol/L of glucose at 37°C for 30 min. After that, the INS-1 cells were stimulated with either 3.3 or 16.7 mmol/L of glucose for 1 h. The medium was collected, and insulin was measured using the rat insulin ELISA kit (Roche Applied Science, Indianapolis, IN, USA) following the manufacturer’s instructions.

### 10. Statistical analysis

Each experiment was conducted in either duplicate or triplicate, and a total of three independent experiments were performed. Data were expressed as mean ± standard deviation (SD). Student’s *t* test was applied for comparisons between two groups, while one-way ANOVA with Tukey’s or Dunnett’s test was applied to compare all groups or specific groups with the control. Statistical analyses were conducted using SPSS 19.0 statistical software (SPSS Inc. USA). *P* values less than 0.05 were considered to be statistically significant.

## Results

### 1. Oxidative stress increases MMP-2 expression and activity in INS-1 cells

**Figure 1:**
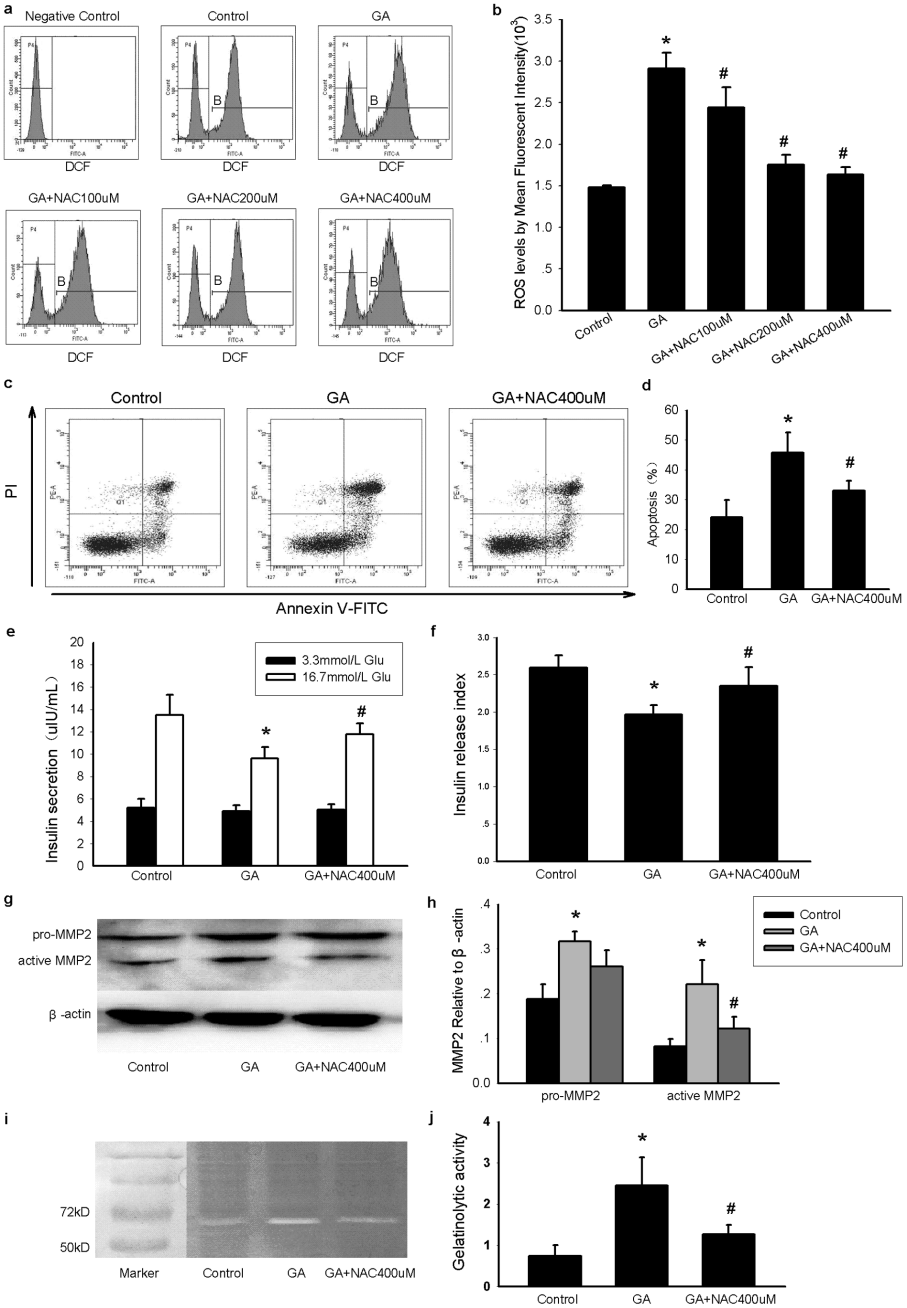
Oxidative stress increases MMP2 expression and activity in INS-1 cells. INS-1 cells were treated with GA (200 mg/L) or BSA (200 mg/L) for 24 h. Antioxidant NAC was dissolved in sterile double-distilled water and used in different final concentrations with GA (200 mg/L). The antioxidant effect of NAC was measured by DCFH-DA through flow cytometer. The concentration with better antioxidant effect was used for following experiments. (a, b) The level of cytosolic ROS in different groups. The mean fluorescence intensity (MFI) of DCF is used to measure cellular ROS levels. BSA–treated cells without DCFH-DA incubation serve as negative control. (c) Apoptotic cells in different groups. Annexin V positive cells (including Annexin V single positive population and the Annexin V and PI double positive population) representing apoptotic cells were mainly located in the right upper quadrant and right lower quadrant. (d) Average apoptotic rate in the three groups. (e) Insulin secretion of INS-1 cells stimulated with 3.3 mmol/L glucose or 16.7 mmol/L glucose in the different groups. *P<0.05 vs the corresponding control group; ^#^ *P<0.05 vs* the corresponding GA group. (f) Insulin release index (IRI) of each group. The IRI was adopted as an index to estimate the insulin secretion function of INS-1 cells. IRI = insulin concentration of high glucose stimulation (16.7 mmol/L)/insulin concentration of low glucose stimulation (3.3 mmol/L). (g, h) Protein expression of MMP2 in different groups, including both pro-MMP2(∼72 kD) and active MMP2(∼62 kD). (i, j) The gelatinolytic activity of MMP2 in different groups. The white band in gray background was the location of MMP2, band intensity was used to represent the activity of MMP2 in each group. Data are shown as means ± SD, *P<0.05* indicates a statistically significant difference. *P<0.05 vs the control group; ^#^ *P<0.05 vs* the GA group.

AGE (GA) treatment significantly increased cytosolic ROS levels, indicating that oxidative stress occurred by this treatment (Fig. 1a and 1b). In addition, apoptosis and insulin secretion dysfunction were also observed after GA treatment (Fig. 1c–f). Interestingly, the protein levels of MMP2 were also elevated in GA-treated cells (Fig. 1 g–h). Moreover, gelatinolytic activity was also increased in INS-1 cells exposed to GA (Fig. 1i and 1j).

However, the antioxidant NAC treatment sharply decreased intracellular ROS level with dose-dependent effect. The cytosolic ROS level even declined near to basal level (control group) when co-treated with GA and 400 uM NAC. Correspondingly, the active MMP2 expression and activity were significantly decreased under NAC treatment (400 uM) compared to GA-treatment group, which further confirmed the effects of oxidative stress on intracellular MMP2 expression and activity. Besides, there was also a decline in INS-1 cell apoptosis and a recovery of insulin secretion function with antioxidant NAC treatment. These results indicated that MMP2 might contribute to the oxidative stress damage in INS-1 cells.

### 2. Forced expression of MMP2 increases apoptosis and leads to dysfunctional insulin secretion in INS-1 cells

**Figure 2:**
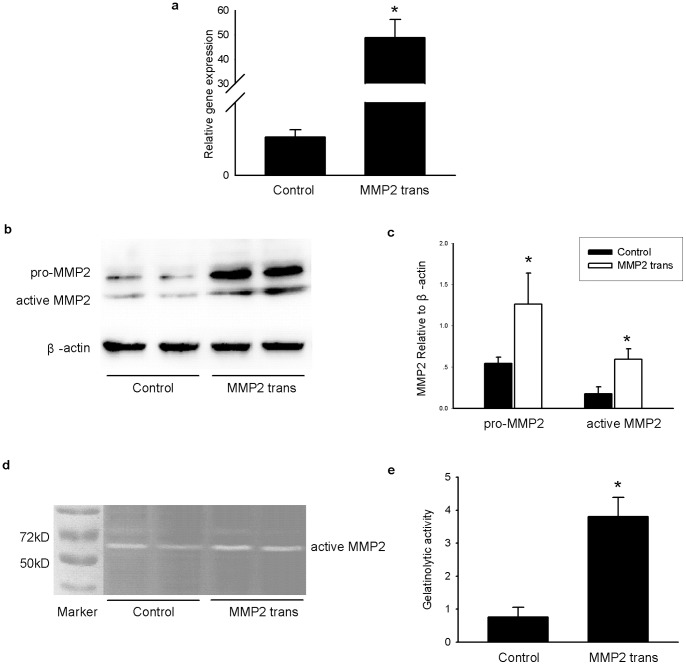
Forced expression of MMP2 in transient transfection of INS-1 cells groups. INS-1 cells were transfected with 1.6 µg MMP2 recombination plasmids and 4 µL Lipofectmine 2000 in each well. An empty vector served as control. After 48 h incubation, the mRNA, protein and gelatinolytic activity of MMP2 were evaluated. (a) Relative mRNA level of MMP2 after 48 h transfection in each group. (b, c) Protein expression (including pro-MMP2 and active MMP2) after 48 h of transfection in each group. (d, e) Gelatinolytic activity of MMP2 after 48 h of transfection in each group. Data are shown as means ± SD, *P<0.05* indicates a statistically significant difference. *P<0.05 vs the control group.

To determine if forced expression of MMP2 is sufficient to mimic the effects of GA treatment, full-length pro-MMP2 cDNA was overexpressed in INS-1 cells. Increased expression levels of MMP2 at the mRNA and protein levels were confirmed (Fig. 2a–c) and MMP2 gelatinolytic activity was also elevated (Fig. 2d, 2e).

**Figure 3:**
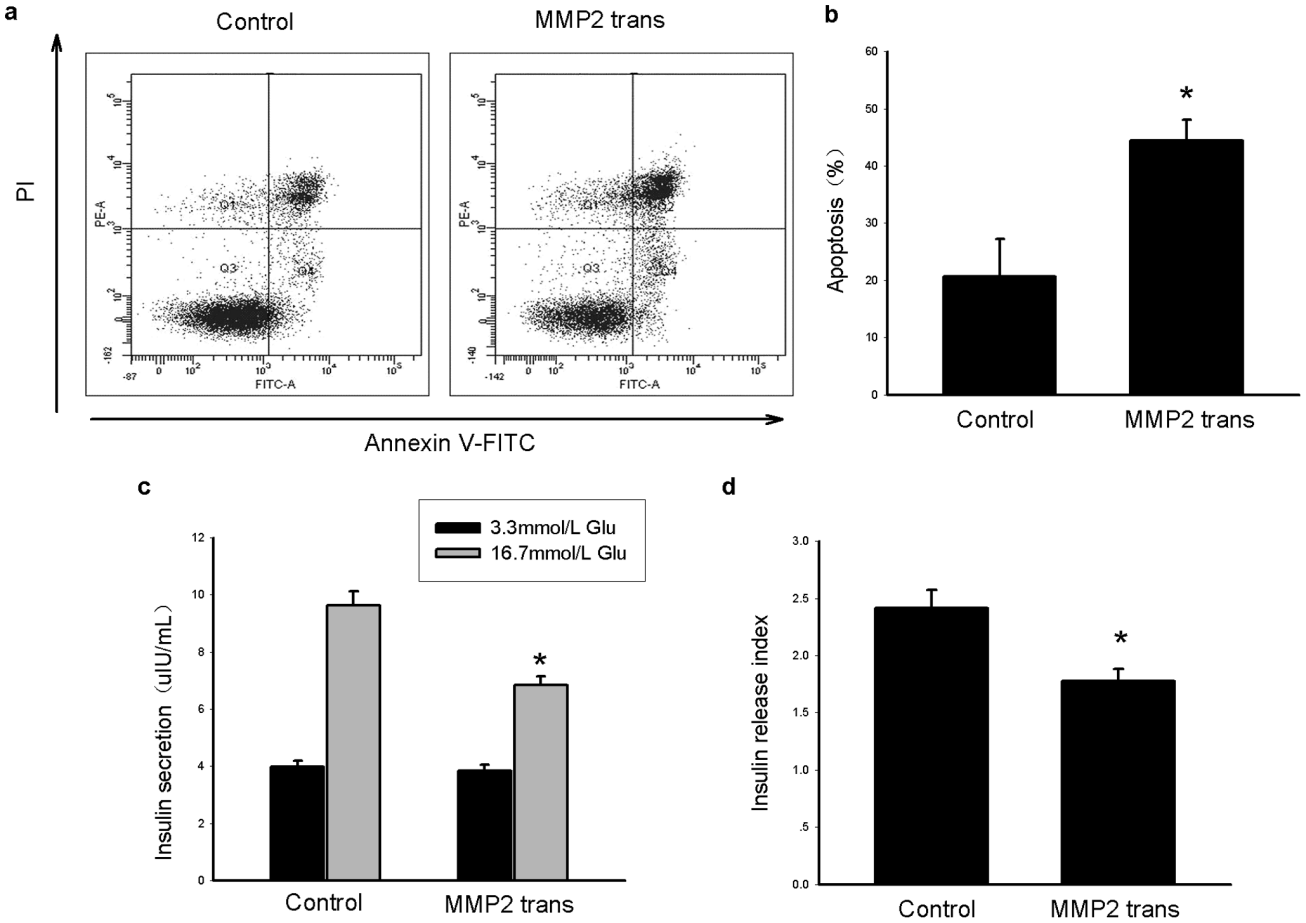
Forced expression of MMP2 increases apoptosis and leads to reduced insulin secretion in INS-1 cells. INS-1 cells were transfected with 1.6 µg MMP2 recombination plasmid and 4 µL Lipofectmine 2000 in each well. An empty vector served as control. The apoptosis and insulin-secretion function of INS-1 cells were evaluated in each group after 48 h of incubation. (a) Apoptotic cells in the control group. Annexin V positive cells (including Annexin V single positive population and the Annexin V and PI double positive population) representing apoptotic cells were mainly located in the right upper quadrant and right lower quadrant. (b) Average apoptotic rate of each group. (c) Insulin secretion stimulated under 3.3 mmol/L or 16.7 mmol/L glucose in each group. *P<0.05 compared to the corresponding control group. (d) Insulin release index (IRI) of each group. Data are shown as means ± SD, *P<0.05* indicates a statistically significant difference. *P<0.05 vs the control group.

MMP2 overexpression increased the apoptosis rate of INS-1 cells by at least two fold (Fig. 3a, 3b). Although the basal insulin secretion (3.3 mmol/L glucose stimulation) in INS-1 cells was not affected by MMP2 overexpression (Fig. 3c), insulin secretion declined when exposure to high amounts of glucose (16.7 mmol/L) (Fig. 3c). This result was consistent with a decreased insulin release index (IRI) (Fig. 3d).

### 3. Inhibition of MMP2 decreases apoptosis and attenuates dysfunctional insulin secretion caused by oxidative stress in INS-1 cells

**Figure 4:**
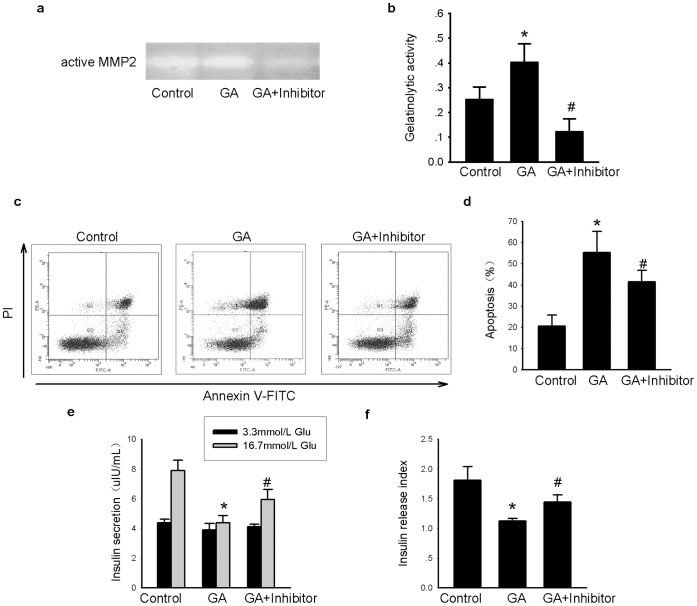
Inhibition of MMP2 decreases apoptosis and attenuates insulin secretion dysfunction caused by oxidative stress in INS-1 cells. INS-1 cells were treated with GA (200 mg/L) or BSA (200 mg/L) for 24 h. MMP2 inhibitor (OA-Hy) was dissolved in DMSO and used at a final concentration of 20 µmol/L with GA for 24 h. (a, b) The gelatinolytic activity of MMP2 in different groups. (c) Apoptotic cells in different groups. Annexin V positive cells (including Annexin V single positive population and the Annexin V and PI double positive population) representing apoptotic cells were mainly located in the right upper quadrant and right lower quadrant. (d) Average apoptotic rate in the three groups. (e) Insulin secretion of INS-1 cells stimulated with 3.3 mmol/L glucose or 16.7 mmol/L glucose in the different groups. *P<0.05 vs the corresponding control group; ^#^ *P<0.05 vs* corresponding GA-treated group (f) Insulin release index (IRI) of each group. Data are shown as means ± SD, *P<0.05* indicates a statistically significant difference. *P<0.05 vs the control group; ^#^ *P<0.05 vs* GA-treated group.

To determine if the up-regulation of MMP2 is necessary for the effects of GA treatment, a MMP2 inhibitor, OA-Hy, was used. The effect of OA-Hy as a MMP2 inhibitor was confirmed by gelatin zymography (Fig. 4a, 4b). OA-Hy significantly decreased apoptosis in GA-treated cells (Fig. 4c, 4d). In addition, insulin secretion of INS-1 cells increased with the administration of the MMP2 inhibitor after treatment with GA (Fig. 4e, 4f).

**Figure 5:**
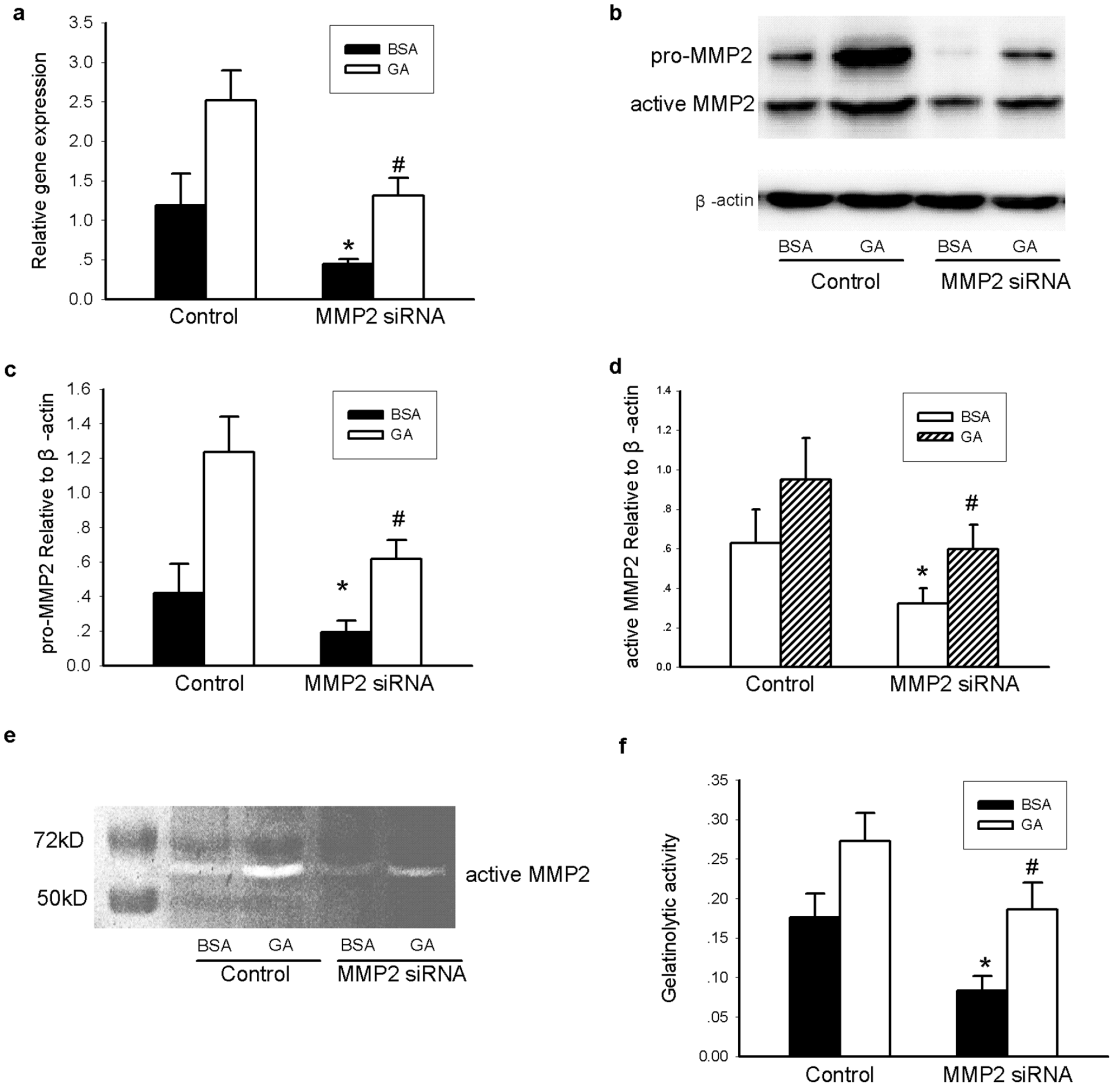
Transient transfection of MMP2 siRNA decreased intracellular MMP2 expression and gelatinolytic activity in INS-1 cells. INS-1 cells of each well were transfected with 40 pmol MMP-2 siRNA by 2 µL Lipofectmine 2000 in each well. A scrambled siRNA was used as control. 48 h after siRNA transfection, INS-1 cells were then treated with GA (200 mg/L) or BSA (200 mg/L) for another 24 h. (a) mRNA expression of MMP2 in different groups; (b–d) Protein expression (including pro-MMP2 and active MMP2) in each group; (e, f) Gelatinolytic activity of MMP2 in each group. Data are shown as means ± SD, *P<0.05* indicates a statistically significant difference. * *P<0.05*, ^#^ *P<0.05* vs corresponding control.

**Figure 6:**
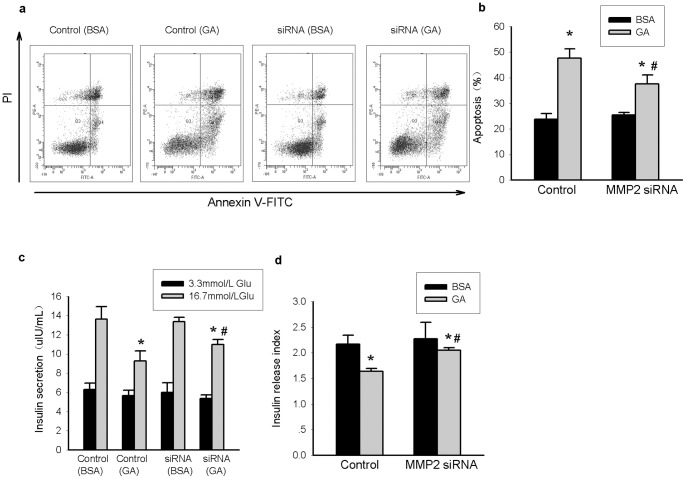
Decreased expression of MMP2 attenuates beta cell dysfunction caused by oxidative stress in INS-1 cells. INS-1 cells were transfected with 40 pmol MMP-2 siRNA. A scramble siRNA was used as control. 48 h after siRNA transfection, INS-1 cells were then treated with GA (200 mg/L) or BSA (200 mg/L) for another 24 h. (a) Apoptotic cells in different groups. Annexin V positive cells (including Annexin V single positive population and the Annexin V and PI double positive population) representing apoptotic cells were mainly located in the right upper quadrant and right lower quadrant. (b) Average apoptotic rate of each group. (c) The insulin secretion stimulated under 3.3 mmol/L glucose or 16.7 mmol/L glucose in each group. *P<0.05 vs BSA-treated subgroup under 16.7 mmol/L glucose stimulation.^ #^ *P<0.05 vs* corresponding control (treated with GA) under 16.7 mmol/L glucose stimulation. (d) Insulin release index (IRI) of each group. Data are shown as means ± SD, *P<0.05* indicates a statistically significant difference. *P<0.05 vs BSA-treated subgroup, ^#^ *P<0.05 vs* corresponding control (treated with GA).

To exclude non-specific effects of the MMP2 inhibitor OA-Hy, MMP2 siRNA was used. Decreased MMP2 mRNA and protein expression levels and decreased MMP2 activity confirmed that this siRNA was effective in INS-1 cells (Fig. 5). Consistent with our results with the MMP2 inhibitor, MMP2 siRNA ameliorated insulin secretion dysfunction and apoptosis of INS-1 cells induced by oxidative stress (Fig. 6), further confirming that MMP2 is required for the effects of GA treatment in INS-1 cells.

## Discussion

The major findings of the present study are as follows. First, oxidative stress increases MMP-2 expression and activity in INS-1 cells. Second, forced expression of MMP2 increases apoptosis and leads to dysfunctional insulin secretion in INS-1 cells. Finally, inhibition of MMP2 either by pharmacologic means or siRNA decreases apoptosis and attenuates insulin secretion dysfunction caused by oxidative stress in INS-1 cells. To the best of our knowledge, this study shows for the first time that MMP2 controls pancreatic beta cell injury induced by oxidative stress.

MMP2, a gelatinase family MMP, is expressed in almost all cells and can degrade collagen IV and other ECM components. Recent findings have indicated that intracellular MMP2 might play an important role in cell injury, especially caused by oxidative stress. ROS has been reported to increase pro-MMP2 release in vascular smooth cells through NAD(P)H oxidase in response to mechanical stretch [19]. In addition, MMP2 in retinal capillary cells was activated by superoxide in the development of diabetic retinopathy [20]. Moreover, MMP2 has been shown to be expressed in pancreatic islets of Zucker diabetic fatty (ZDF) rats, and the expression and activity of MMP2 were increased along with the onset of islet dysfunction and diabetes [17]. All this evidence strongly suggests that MMP2 may also be involved in pancreatic beta cell injury induced by increased oxidative stress. Here, we show that oxidative stress increases MMP-2 expression and activity in INS-1 cells while antioxidant NAC attenuates this increase in MMP2 expression/activity by blocking ROS level, indicating that increased expression of MMP2 is consistent with increased oxidative stress in INS-1 cells. Actually, our results from western blot show that there is only a slight attenuation in the expression of pro-MMP2 without statistical significant, whereas the expression of active MMP2 in NAC-treatment group drastically decreases compared to GA-treatment group. Although unproven, we assume that enhanced oxidative stress might also aggravate cleavage of proenzyme into active form of MMP2 in addition to regulating expression of intracellular MMP2. Giving further support to this assumption, similar findings have also been reported that the increase in active MMP2 other than pro-MMP2 has been partially reversed in aortic tissue of diabetic rats treated with antioxidant [21]. Meanwhile, forced expression of MMP2 mimics the effects of oxidative stress induced in INS-1 cells in our study, suggesting that MMP2 overexpression is sufficient to mimic the effects of oxidative stress-induced damage in INS-1 cells. PD-166793, a broad-spectrum MMP inhibitor, has been shown to prevent female ZDF rats on a high-fat diet from beta-cell dysfunction and diabetes, though the subfamily of MMP responsible for this effect is unclear [17]. In the present study, we show that a MMP2-selective inhibitor OA-Hy can improve INS-1 cell insulin secretion function and reduce apoptosis induced by oxidative stress. Moreover, specific down-regulation of MMP2 by MMP2 siRNA shows the same beneficial effects as the MMP2 chemical inhibitor. These results indicate that up-regulation of MMP2 is necessary for the effects of GA treatment in pancreatic beta cells.

Still we have no conclusions on the mechanism of beta cell dysfunction induced by AGE. Recent evidence suggests that there exist several pathways which may mediate AGE-induced beta cell dysfunction. It was reported that AGE could impair insulin synthesis and secretion of INS-1 cells through enhancing glycogen synthase kinase-3 (GAK-3) activation to further decrease PDX-1 expression other than increasing ROS production [22]. Another proapoptotic mechanism of AGE was also discovered that autophagy deficiency as well as increased endoplasmic reticulum stress and oxidative stress, resulting in nuclear factor-κB (p65)-iNOS-caspase-3 cascade activation, contributed to AGE-mediated pancreatic beta cell apoptosis and dysfunction [23]. The results in our study showed that inhibiting intracellular MMP2 activity could significantly reduce GA-induced beta cell apoptosis and improve insulin secretion, although not totally reversed, which suggested that MMP2 played an important role in one of oxidative stress-activated pathways induced by AGE. However, the downstream target of MMP2 responsible for the pancreatic beta cell dysfunction induced by oxidative stress remains unclear. In cardiomyocytes, oxidative stress-induced MMP2 expression is due to the activation of activator protein-1 (AP-1) and/or nuclear factor-kappa B (NF-κB) [24], [25]. In addition, whether intracellular MMP2 serves as a protease or only as a signaling protein to induce apoptosis, or both, in pancreatic beta cell oxidative injury, is worthy of further study. It has been reported that intracellular MMP2 was responsible for the loss of mitochondrial potential and increased mitochondrial membrane permeability in retinal endothelial cells, which induced the translocation of Bax and cytochrome C from the mitochondria to the cytoplasm to activate intracellular apoptotic mechanisms [26], [27]. Nevertheless, this study gives direct evidence that MMP2 is necessary and sufficient for pancreatic beta cells injury induced by oxidative stress.

In conclusion, this study shows that MMP2 expression and intracellular activity are increased by oxidative stress, contributing to cellular dysfunction and apoptosis in INS-1 cells after AGE challenge. Inhibition of intracellular MMP2 may represent a new therapeutic approach for the protection of pancreatic beta cells from oxidative injury and the prevention of diabetes.
